# The double-positive cells in the tumor microenvironment

**DOI:** 10.1515/jtim-2026-0002

**Published:** 2026-02-13

**Authors:** Xinmiao Long, Yinfei Du, Yinan Li, Fan Guan, Shiyi Wang, Meng Huang, Minghua Wu

**Affiliations:** The Affiliated Cancer Hospital of Xiangya School of Medicine, Central South University/Hunan Cancer Hospital, Central South University, Changsha, Hunan Province, China; The Key Laboratory of Carcinogenesis of the Chinese Ministry of Health, The Key Laboratory of Carcinogenesis and Cancer Invasion of the Chinese Ministry of Education, Cancer Research Institute, Central South University, Changsha, Hunan Province, China; Department of Neurosurgery, Xiangya Hospital, Central South University, Changsha, Hunan Province, China; FuRong Laboratory, Changsha, Hunan Province, China

**Keywords:** tumor-associated macrophages, tumor microenvironment, immune reprogramming

## Abstract

The tumor microenvironment substantially influences cancer progression by mediating complex interactions between immune cells, fibroblasts, endothelial cells, and mesenchymal cells. Recent studies have identified a critical component of this ecosystem, double-positive cells (DPCs), which are characterized by simultaneously expressing two markers that are traditionally confined to completely different cell lineages or cell types. In this review, we demonstrated DPCs' formation principles, characterization, classification, functions, and clinical significance. We underscore the multifaceted contributions of DPCs in enhancing tumor invasiveness, facilitating immune evasion, and promoting drug resistance. Understanding the significance of targeting DPCs could open new avenues for therapeutic interventions in cancer treatment.

## Introduction

Contemporary advancements in single-cell multiomics technology have revealed that tumor development and metastasis are governed and sustained by a complex ecosystem, the tumor microenvironment (TME). The TME, orchestrated by cancer cells, encompasses a diverse array of noncancerous cells embedded within an altered extracellular matrix.^[1]^ Dynamic interactions within the TME not only provide essential nutrients and growth factors crucial for cancer cell proliferation but also coordinate signaling pathways that facilitate immune evasion and promote tumor progression and dissemination.^[2,3]^ The TME involves intricate interactions between various cell types, including immune, stromal, endothelial, and diverse tissue-resident cells. The composition and functional dynamics of the TME exhibit considerable variability and are influenced by factors such as tumor origin, cancer cell characteristics, disease stage, and patient-specific attributes.^[4,5]^

Recently, the discovery of double-positive cells (DPCs) has attracted considerable attention. Although the cellular composition of the TME is highly heterogeneous, certain cell subsets and gene expression patterns remain conserved *in vivo*.^[6]^ For example, the immune cell marker cluster of differentiation 45 (CD45) is typically not expressed in tumor or stromal cells. Similarly, the myeloid cell marker CD14 is generally not found on T cells. In lineage-specific contexts such as T cells, it is commonly believed that immature T cells undergo negative selection and do not co-express CD4 and CD8. However, in the TME, markers that are conventionally restricted to entirely distinct lineages or cell types have been observed to co-exist within the same cells.^[7, 8, 9]^ We define these cells as DPCs. DPCs are a novel cell type characterized by the simultaneous expression of two distinct cellular markers. These cells arise through diverse mechanisms and play multifaceted roles in tumor immunity.

The spontaneous fusion of cancer cells was first proposed as early as 1911, with the initial hypothesis suggesting that such hybrid cells might represent one of the origins of cancer.^[10]^ In 1965, Scaletta *et al*. successfully conducted *in vitro* hybridization between normal cells and tumor cells, marking the beginning of research into DPCs. Subsequent studies demonstrated the potential of human leukocytes to fuse with tumor cells and form DPCs. Darlington observed that cells resulting from the fusion of mouse hepatocellular carcinoma cells and human leukocytes were capable of secreting both mouse and human serum albumin.^[11]^ DPCs formed by the fusion of melanoma cells and macrophages exhibited enhanced metastatic potential.^[12]^ In recent years, DPCs co-expressing immune and tumor cell markers have been widely reported.^[8,13]^ In the 1980s, double-positive T cells were first identified in peripheral blood.^[14]^ In 1998, Bagot *et al*. isolated CD4⁺CD8⁺ T cells from cutaneous T cell lymphoma and demonstrated that this cell population was present exclusively at the tumor site.^[15]^ More recent studies suggest that CD4⁺CD8⁺ T cells constitute a component of the TME.^[16,17]^ However, many studies generated DPCs through artificial *in vitro* hybridization to explore their functions. As researchers gradually recognized the heterogeneity and complexity of the TME, this approach no longer adequately reflects the true *in vivo* status of DPCs in patients and their contribution to the TME. In addition to DPCs formed by hybridization with tumor cells, a large number of DPCs with unique characteristics in the TME have been extensively characterized with the widespread use of single-cell sequencing.^[18,19]^ This has led to a renewed understanding of the importance of DPCs in the TME. Therefore, we conducted a comprehensive literature review of recent reports on DPCs within the TME. This review critically analyzes and summarizes the current literature to elucidate the mechanisms and specific functions of DPCs in the TME. Its contribution to either promoting or inhibiting tumor progression has been explored, and potential applications of DPCs in innovative cancer therapies have been proposed.^[20]^

## Characterization and diversity of DPCs

DPCs are unique cell types that simultaneously express two marker genes which are traditionally confined to completely different cell lineages or cell types. In light of this, we have classified DPCs based on the signature genes they express, which represent the marker genes of different cell types or lineages, in order to better distinguish the relevant cells. For example, if a group of DPCs expresses both CD45 (an immune cell marker) and epithelial cell adhesion molecule (EPCAM, a tumor cell marker), we refer to it as an immune-tumor cell.

**Figure 1:**
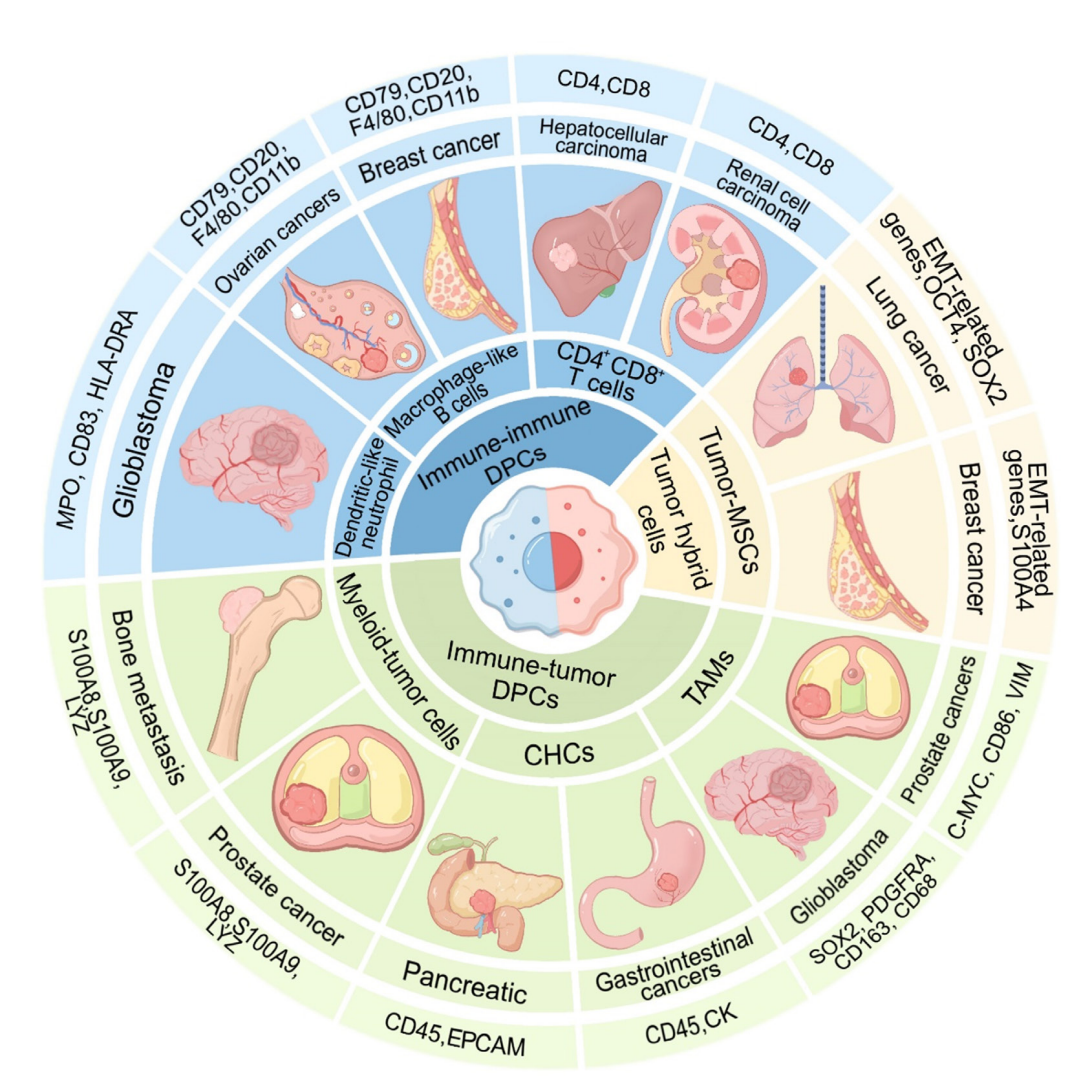
The classification, distribution, and marker genes of DPCs. DPCs can be categorized into three major types: Immune-tumor DPCs, immune-immune DPCs, and tumor hybrid cells. Among them, immune-tumor DPCs can be further divided into myeloid-tumor cells, CHCs, and TAMs. Immune-immune DPCs can be further subdivided into CD4^+^CD8^+^ T cells, CD14^+^CD8^+^ T cells, and macrophage-like B cells. Tumor hybrid cells are composed primarily of tumor-MSCs. These different DPCs are distributed across various types of cancers, and the genes used to identify them are listed in detail. Macrophage-like B cells: DPCs that simultaneously express macrophage and B cell marker genes. Dendritic-like neutrophils: DPCs that simultaneously express neutrophil and dendritic cell marker genes. DPCs: double-positive cells; CD: cluster of differentiation; MSCs: mesenchymal stroma/stem-Like cells; TAMs: tumor-associated macrophages; CHCs: circulating hybrid cells; CK: cytokeratin; MPO: myeloperoxidase; HLA-DRA: HLA class II histocompatibility antigen, DR alpha chain; S100A8: S100 calcium binding protein A8; LYZ: lysozyme; EPCAM: epithelial cell adhesion molecule; SOX2: SRY (sex determining region Y)-box 2; PDGFRA: platelet-derived growth factor receptor alpha; C-MYC: MYC proto-oncogene, bHLH transcription factor; VIM: vimentin; EMT: epithelial-mesenchymal transition; OCT4: octamer-binding transcription factor 4.

DPCs are substantially diverse and are classified into immune-tumor DPCs, immune-immune DPCs, and tumor hybrid cells. Each category has distinct characteristics and functions that contribute to overall cellular diversity in the TME (Figure 1 and Table 1). Focusing on these complex DPCs can help us understand the diversity of the TME, including intercellular communication, signaling pathways, and cellular state dynamics.

**Table 1:** Comparative diversity of DPCs in different tumor types

Cell type		Cancer type	Markers	Function in the TME	Reference
Immune-tumor DPCs	CHCs	Pancreatic	CD45, EPCAM	Enhanced EMT phenotype proliferative and tumorigenic capacities immune evasion drug resistance immunosuppression	Zhang *et al*.^[150]^
		Ovarian cancer	CD45, CXCR4, EPCAM		Akhter *et al*.^[8]^
		Non-small cell lung cancer	CD14, CK, CD45, EPCAM		Manjunath *et al*.^[151]^, Aguirre *et al*.^[152]^
		Oral cavity cancer	CD45, CK		Henn *et al*.^[153]^
		Breast cancer	CD45, CK, CD163		Lustberg *et al*.^[154]^, Shabo *et al*.^[155]^
		Gastrointestinal cancers	CD45, CK		Walker *et al*.^[156]^
	Myeloid-tumor cells	Nasopharyngeal carcinoma	EPCAM, HLA-DRB2	Immunosuppression tumorigenic capacities drug resistance	Jin *et al*.^[24]^
		Prostate cancer Bone metastasis	KRT8, KRT18, S100A8, S100A9, LYZ	Immunosuppression enhanced EMT phenotype and tumorigenic capacities	Ye *et al*.^[30]^
	TAMs	GBM	SOX2, PDGFRA, CD163, CD68	Immunosuppression	Wu *et al*.^[21]^ Cao *et al*.^[22]^ Long *et al*.^[25]^
		Prostate cancer	C-MYC, CD86, VIM	Immune evasion immunosuppression enhanced EMT phenotype	Chou *et al*.^[23]^
Immune-immune DPCs	CD4^+^CD8^+^ T cells	Urological cancers	CD4, CD8	Enhanced Th2 cytokine production	Bohner *et al*.^[43]^
		Hepatocellular carcinoma		immunosuppression or enhanced cytotoxicity	Zheng et al.^[16] ^Wang *et al*.^[17]^
		Renal cell carcinoma		High levels of inhibitory receptors	Menard *et al*.^[44]^
		Melanoma Lung cancer		Immunosuppression or enhanced cytotoxicity	Schad *et al*.^[35]^
	CD14^+^CD8^+^ T cells	Liver	CD14, CD8	High IL-10 and IL-2 production enhanced anti-tumor function	Pallett *et al*.^[7]^
	Macrophage-like B cells	Breast cancers ovarian cancers	CD79, CD20, F4/80, CD11b	Immunosuppression	Chen *et al*.^[18]^
	Dendritic-like neutrophils	GBM	MPO, CD83, HLA-DRA	Enhanced antigen presentation capability	Lad *et al*.^[19]^
Tumor hybrid cells	Tumor-MSCs	lung cancer	EMT-related genes, OCT4, SOX2	Enhanced metastatic capacity characteristics of cancer stem cells	Zhang *et al*.^[55]^
		Breast cancer	EMT-related genes, S100A4		Melzer *et al*.^[56]^

DPCs, double-positive cells; CHCs, circulating hybrid cells; EMT, epithelial-mesenchymal transition; GBM, glioblastoma; TAM, tumor-associated macrophages; CD, cluster of differentiation; CK, cytokeratin; IL, Interleukin; MSCs, mesenchymal stroma/stem-Like cells; MPO: myeloperoxidase; HLA-DRA: HLA class II histocompatibility antigen, DR Alpha Chain; S100A8: S100 calcium binding protein A8; LYZ: lysozyme; EPCAM: epithelial cell adhesion molecule; SOX2: SRY (sex determining region Y)-box 2; PDGFRA: platelet-derived growth factor receptor alpha; C-MYC: MYC proto-oncogene, bHLH transcription factor; VIM: vimentin; EMT: epithelial-mesenchymal transition; OCT4: octamer-binding transcription factor 4.

### Immune-tumor DPCs

Immune-tumor DPCs refer to cells that co-express marker genes of both tumor cells and immune cells. They are widely present in the TME and play a crucial role in tumor initiation and progression. Both the teams of Cao *et al*. and Wu *et al*. have identified the presence of DPCs in glioblastoma (GBM). These cells expressed macrophage marker genes (CD163, Fc gamma receptor IIa [FCGR2A], Macrophage scavenger receptor 1[MSR1] and Integrin subunit alpha X [ITGAX]) and harbored GBM-derived mutations or glioma biomarkers (glial fibrillary acidic protein [GFAP] and platelet-derived growth factor receptor alpha [PDGFRA]).^[21,22]^ Chou *et al*. reported that DPCs were formed by low CD47 signal-mediated incomplete digestion of tumor cells by macrophages.^[23]^ Following the phagocytosis of enhanced green fluorescent protein (EGFP)-labeled tumor cells, these macrophages give rise to CD86⁺EGFP⁺ DPCs, which is indicative of their functional reprogramming and acquisition of tumor-derived components.^[23]^ Akhter *et al*. reported that DPCs expressed both EPCAM and CD45 in the ascites of patients with epithelial ovarian cancer, demonstrating drug resistance and evasion of natural killer (NK) cell-mediated immune surveillance.^[8]^ Jin *et al*. identified DPCs in nasopharyngeal carcinoma that co-express EPCAM and human leukocyte antigen-D related beta 2 (HLA-DRB2) and exhibited both epithelial and immune features *via* single-cell RNA sequencing (scRNA-seq). These cells display immunosuppressive properties and possess the ability to repress interferon gamma (IFN-γ) production by T cells.^[24]^ Long *et al*. reported a group of DPCs in GBM patients with cytomegalovirus (CMV) infection that concurrently express tumor cell marker genes (SRY-box transcription factor 2 [SOX2]) and macrophage marker genes (CD68). These cells are highly infiltrative and are associated with poor patient prognosis.^[25]^ In summary, numerous DPCs exhibit both immune cell and tumor cell characteristics within the TME. These cells mainly display immunosuppressive properties, promote tumor progression, and enhance resistance to therapy.

Tumor cell migration, extravasation, and metastasis are critical steps in tumor progression.^[26, 27, 28]^ A growing body of evidence indicates that immune-tumor DPCs are abundant during these processes and play crucial roles.^[29]^ Gast *et al*. revealed that circulating hybrid cells (CHCs) with hematopoietic and epithelial characteristics acquire macrophage-associated features and increase their migratory and invasive capabilities.^[13]^ Ye *et al*. discovered a novel type of DPCs at bone metastasis sites that expresses the characteristics of both myeloid and tumor cells, has considerably increased proliferation rates and metastatic potential, and exhibits an enhanced epithelial-mesenchymal transition (EMT) phenotype and increased tumorigenicity and chemotherapy resistance.^[30]^ Ali *et al*. had comprehensively characterized the functional features and potential origins of immune-tumor DPCs using single-cell and spatial transcriptomics.^[31]^ Cytokeratin (CK) is a specific marker of epithelial cancer cells, and its content can be used to characterize tumorigenesis, progression, and metastasis.^[32,33]^ Clinically, the presence of CHCs is often detected by examining the co-expression of CK and other immune markers within a single cell.^[34]^

### Immune-immune DPCs

Immune-immune DPCs represent a population of DPCs that express characteristic genes of multiple distinct types of immune cells. In recent years, an increasing number of CD4⁺CD8⁺ T cells have been identified in the TME and inflamed tissues.^[35]^ T cells typically undergo a series of positive selections in the thymus, transitioning from double-positive CD4^+^CD8^+^ T cells to single-positive CD4^+^ or CD8^+^ T cells, marking the central stage of T cell development. Therefore, it is widely accepted that double-positive CD4^+^CD8^+^ T cells represent a transient developmental state and are only present in the thymus.^[36, 37, 38]^ Consequently, mature CD4^+^CD8^+^ T cells are frequently regarded as anomalies and are often dismissed as either cell doublets or erroneous cells that have evaded thymic selection. However, a rising number of studies have identified the presence of double-positive T cells outside the thymus, where they participate in immune responses.^[39,40]^ Double-positive T cells have been identified in a variety of diseases. For example, in the peripheral blood of human immunodeficiency virus (HIV)-infected patients, double-positive T cells with strong proliferative capabilities have been detected, and these cells exhibit multifunctionality when stimulated with specific HIV antigens.^[41]^ In patients with systemic sclerosis, Interleukin-4 (IL-4) secreted by double-positive T cells was found to enhance the extracellular matrix deposition by fibroblasts.^[42]^ This suggests that double-positive T cells can be widely involved in the pathogenesis and progression of diseases. Numerous studies have also shown that double-positive T cells can be a component of the TME, participating in the remodeling of the tumor immune microenvironment. Bohner *et al*. revealed the enrichment of CD4^+^CD8^+^ double-positive T cells in urological cancers, noting their preference for Th2 cell polarization while inhibiting Th1 cell induction.^[43]^ Zheng *et al*. analyzed the trajectory and function of CD4^+^CD8^+^ double-positive T cells in hepatocellular carcinoma, discovering elevated programmed cell death protein 1 (PD-1) levels.^[16]^ Notably, Menard *et al*. demonstrated that CD4^+^CD8^+^ double-positive T cells are abundantly expanded in kidney cancer and express high levels of immunosuppressive receptors, such as PD-1 and T-cell immunoglobulin and mucin-domain containing-3 (TIM-3).^[44]^ Wang *et al*. reported a significant increase in the number of tumor-associated CD4^+^CD8^+^ double-positive T cells, which exhibit stem-like and enhanced cytotoxic characteristics, within the tumor immune microenvironment of patients treated with a novel Arf1 inhibitor.^[17]^ You *et al*. found that the deficiency of Mettl3 in CD4^+^CD8^+^ double-positive T cells disrupts the expression of invariant natural killer T cell (iNKT) cell-related genes, leading to a defect in melanoma resistance.^[45]^ Overall, CD4^+^CD8^+^ double-positive T cells exhibit varying traits within the TME, contributing to immunosuppression and diversity.

The differentiation of immune cells is a tightly regulated process, ensuring that most immune cells express only lineage-specific genes and maintain clear molecular boundaries between cell types.^[46]^ However, recent studies have shown the presence of dual-phenotype immune cells (immune-immune DPCs) that simultaneously exhibit features of multiple immune cell types, which has been experimentally validated. The CD14^+^CD8^+^ T cells that simultaneously express markers of both myeloid and lymphoid lineages have been identified in the liver. These cells exhibit increased turnover, activation, and intrinsic immunomodulatory properties characterized by the production of IL-10 and IL-2.^[7,47]^ Chen *et al*. reported a class of DPSs that simultaneously express macrophage-specific genes CD68, colony stimulating factor 1R (CSF1R), and B cell-specific genes CD79 and CD20, and referred to them as macrophage-like B cells.^[18]^ Research has shown that breast and ovarian cancers cause bone B cell precursors to accumulate in the spleen, where they are converted into B cells that promote metastasis and suppress the immune system.^[18]^ Zhang *et al*. observed CD3^+^CD19^+^ T/ B phenotypic lymphocytes in both humans and mice. In response to antigen-specific stimulation, these dual-phenotype lymphocytes secrete higher levels of IL-2 but lower levels of tumor necrosis factor-α (TNF-α).^[48]^ Wang *et al*. identified a cytotoxic B cell subset (B cell receptor^+^ [BCR^+^] and Granulysin⁺ [GNLY⁺] T cells) that is enriched in children.^[49]^ Lad *et al*. were the first to identify a group of tumor-associated neutrophils expressing major histocompatibility complex (MHC) class II molecules in GBM, originating from the cranial bone marrow (BM).^[19]^ These cells develop from neutrophils (expressing genes such as myeloperoxidase [MPO], CD66b, *etc*.), but also exhibit dendritic cell characteristics (expressing costimulatory ligands CD83/86/40 and MHC II subunits human leukocyte antigen - DR beta 3 /DR alpha, human leukocyte antigen - DP alpha 1 /DP Beta 1 (HLA-DRB3/A, DPA1/B1), and are therefore referred to as dendritic-like neutrophils.^[19]^ Overall, the discovery of immune-immune DPCs has greatly expanded our understanding of the human immune system, and further studies are needed to determine the specific functions of these unique cells.

### Tumor hybrid cells

Mesenchymal stromal/stem-like cells (MSCs) are defined as a pluripotent cell population with self-renewal abilities found in various tissues, or as stem cell-like stromal cells with plasticity.^[50,51]^ A wealth of literature reports that they are also an important component of the TME and play a role in the progression and development of tumors.^[52]^ Tumor hybrid cells are the fusion products of tumor cells and MSCs, and they represent an important type of DPCs within the TME. These cells exhibit diverse characteristics but generally play critical roles in tumor progression, metastasis, and drug resistance.^[53,54]^ Zhang *et al*. reported that cell fusion between lung cancer cells and MSCs enhances the metastatic capacity and characteristics of cancer stem cells through EMT.^[55]^ Melzer *et al*. demonstrated the spontaneous development of new tumor cell populations exhibiting different parental properties after close interaction and subsequent fusion of MSCs with breast cancer cells.^[56]^ Liu *et al*. discovered that the fusion of glioma cells with MSCs can enhance M2 polarization through the m6A modification of CSF1.^[57]^ Interestingly, some studies have reported that tumor hybrid cells formed by the fusion of MSCs with malignant cells exhibit reduced tumorigenicity and exert inhibitory effects on tumor growth, which contradicts the findings mentioned above. Melzer *et al*. demonstrated that tumor hybrid cells formed by the fusion of MSCs with ovarian cancer cells exhibit reduced proliferative capacity and tumorigenicity.^[58]^ Wang *et al*. reported similar phenomena in esophageal cancer.^[59]^ The above results indicate that tumor hybrid cells possess both tumor-promoting and tumor-inhibitory roles, which may be caused by many factors. Firstly, the different types of tumor cells are a major reason for the functional heterogeneity of DPCs. Using the same method, DPCs formed by the spontaneous fusion of ovarian cancer cell line SK-OV-3 with MSCs exhibited weaker tumorigenicity and proliferative capacity compared to those formed by spontaneous fusion of breast cancer cell line MDA-MB-231 with MSCs.^[56,58]^ Secondly, the methodological differences may also affect the identification of the functional characteristics of DPCs. Zhang *et al*. used polyethylene glycol 1450 (PEG1450) for artificial induction of cell hybridization, and Wang *et al*. also chose artificial induction through PEG1500.^[55,59]^ In contrast, Melzer used fluorescently labeled cell lines for spontaneous fusion *via in vitro* co-culture to generate DPCs.^[56,58]^ Notably, Liu *et al*. abandoned cell lines and instead used primary cells for spontaneous fusion *in vitro*, further enhancing the persuasiveness and credibility of the study.^[57]^ Finally, the functional diversity of tumor hybrid cells is not only reflected in the inherent characteristics of the tumor cells themselves but is also closely related to the microenvironment. Liu *et al*. demonstrated that tumor hybrid cells can shape the immunosuppressive microenvironment of GBM by attracting and inducing M2-like polarization of macrophages.^[57]^ Additionally, tumor hybrid cells promote the formation of an immunosuppressive TME through various mechanisms, such as recruiting myeloid-derived suppressor cells, promoting angiogenesis, and differentiating into different cell lineages.^[60]^ In conclusion, tumor hybrid cells with mesenchymal phenotypes have been identified in a wide range of cancers, yet their roles within the TME require further investigation and clarification.

### Other DPCs

Recently, numerous DPCs have also been identified in the normal human body. Yang *et al*. identified immune-featured decidual stromal cells (iDSCs) with a dual profile in their study of endometrial metaplasia. iDSCs exhibit dual immune-stromal cell properties. They facilitate immune cell recruitment and suppression, regulate vascularization, and promote cytolysis at immune cell assemblies and vascularization hubs to establish decidual homeostasis at a later stage.^[61]^ Furthermore, iDSCs can induce cytolysis by activating the granzyme-mediated apoptotic signaling pathway and cancer cell cytolysis. A group of rare low-affinity nerve growth factor receptor (LNGFR)^+^ cells that coexpress endothelial and stromal markers was identified in human fetal and regenerative BM. These cells can generate pluripotent MSCs that rebuild the BM microenvironment after transplantation. This dual functionality underscores their importance in the BM, affecting normal hematopoiesis and diseases, such as leukemia, with potential implications for therapy development in hematologic cancers and BM disorders.^[62]^ Curry *et al*. discovered a population of spiking tumor cells in glioma that exhibit selective characteristics of γ-aminobutyric acid-ergic (GABAergic) neurons and oligodendrocyte precursor cells.^[63]^

In general, immune-tumor DPCs, immune-immune DPCs, tumor hybrid cells, and other DPCs show substantial diversity. The main characteristics of immuno-tumor DPCs are the co-expression of markers for immune and tumor cells. They mainly consist of tumor-associated macrophages (TAMs) formed by macrophage phagocytosis or CHCs formed by fusion. Immune-immune DPCs express multiple immune cell markers and perform diverse functions, broadening our understanding of the human immune system. Tumor hybrid cells are formed mainly by the fusion of tumor cells with epithelial, endothelial, stromal, or MSCs. However, their roles in tumor growth and metastasis remain a subject of ongoing debate. Many other DPCs expressing markers for different types of cells also add to the diversity of DPCs, and further studies are needed.

## Principles underlying the formation of the DPCs

**Figure 2:**
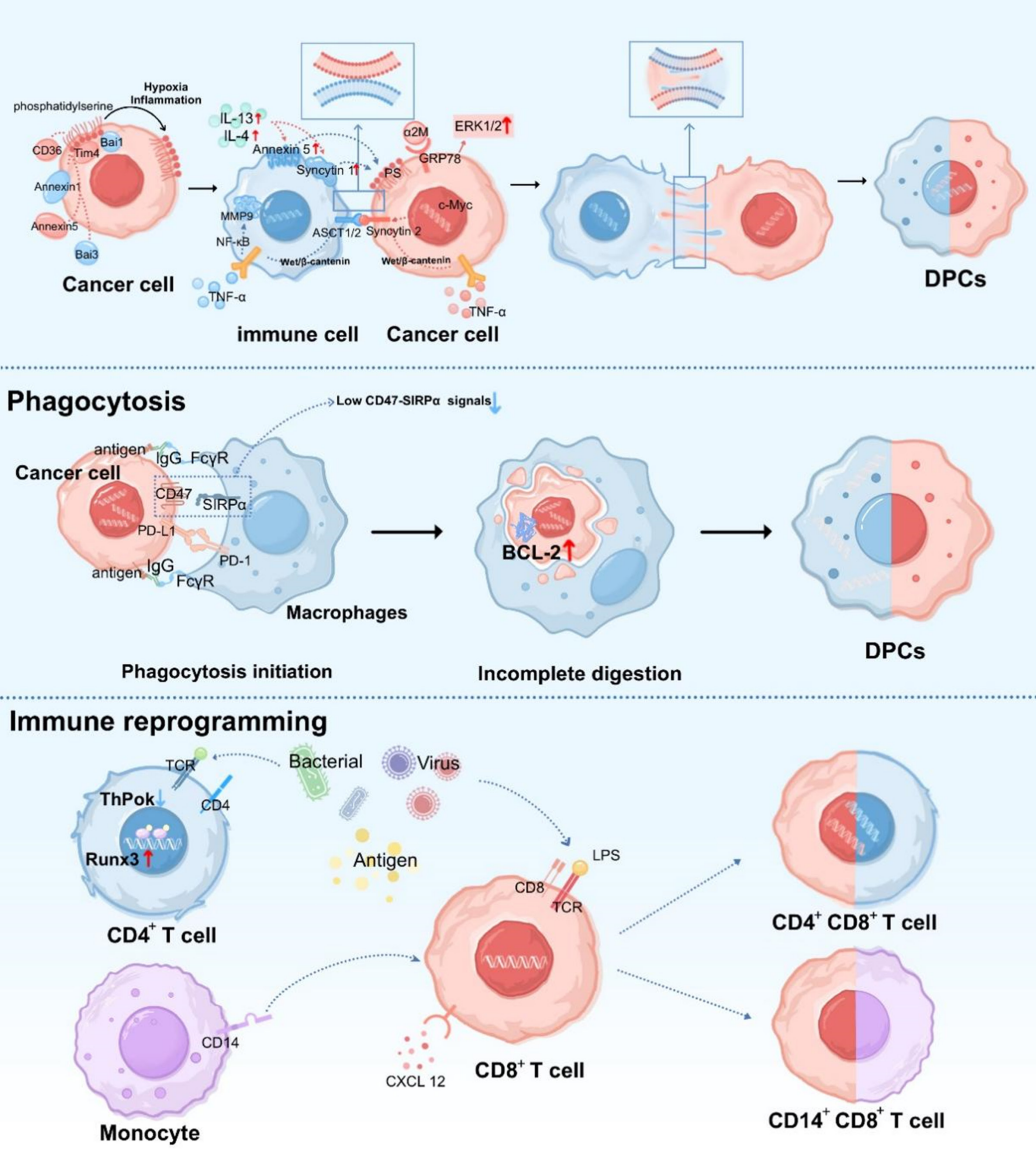
Mechanisms of DPCs formation. Mechanisms of the formation of DPCs involved in cell fusion, phagocytosis and immune reprogramming. Cell fusion: PS interacts with Tim4, Bai1, Bai3, Annexin1, Annexin5, and CD36 on the inner leaflet of the cell membrane. Under conditions such as inflammation and hypoxia, PS becomes externalized. Exogenous IL-4 and IL-13 induce the upregulation of syncytin-1 and membrane-bound Annexin A5, both of which can bind to PS and promote cell fusion. The interaction between α2-macroglobulin and GRP78 activates the ERK1/2 signaling pathway to induce fusion. The interaction between ASCT2 and syncytin-2 also facilitates this process. TNF-α can promote fusion by upregulating MMP9 expression via the Wnt/β-catenin pathway. Local schematic representations showing contact and exchange of the phospholipid bilayers during the fusion process. Phagocytosis: Macrophages interact with various phagocytosis-associated ligand receptors on the surface of tumor cells. Low CD47-SIRPα signaling in cancer cells phagocytosed by macrophages leads to incomplete digestion, as indicated by increased BCL-2 expression, resulting in the formation of DPCs. Immune reprogramming: Antigenic stimulation induces immune cell reprogramming, where CD4^+^ T cells re-express CD8 through altered expression of ThPOK and Runx3. Similarly, CD8^+^ T cells acquire CD4 expression in response to specific stimuli. Monocytes, influenced by CXCL12 and LPS, transfer CD14 to CD8^+^ T cells, resulting in the formation of CD14^+^CD8^+^ DPCs. GRP, glucose-regulated protein; DPCs: double-positive cells; CD: cluster of differentiation; PS: Phosphatidylserine; IL: Interleukin; TNF-α: tumor necrosis factor-α; MMP9: matrix metalloproteinase-9; ThPOK: thymocyte selection-associated high mobility group box protein; Runx3: runt-related transcription factor 3; CXCL12: C-X-C motif chemokine ligand 12; FcγR: Fc gamma receptor; TCR: T cell receptor; PD-L1: programmed death-ligand 1; PD-1: programmed cell death protein 1; Tim4: T Cell immunoglobulin and mucin domain-containing protein 4; Bai1: brain-specific angiogenesis inhibitor 1; GRP78: glucose-regulated protein 78 kDa; ERK1/2: extracellular signal-regulated kinase 1/2; ASCT2: alanine-serine-cysteine transporter 2; SIRP: signal regulatory protein; BCL-2: B-cell lymphoma 2; IgG: immunoglobulin G.

Understanding the origins of DPCs is a critical area in cancer research. Despite interest in their role within the TME, the mechanisms underlying DPC formation remain poorly comprehended. Current hypotheses regarding DPC formation predominantly focus on three mechanisms: Cell fusion, cell phagocytosis, and immune reprogramming (Figure 2).

### Cell fusion

Cell fusion occurs during normal development and the differentiation of specific tissues and encompasses physiological processes, such as fertilization,^[64]^ placental implantation,^[65]^ myogenesis,^[66]^ osteoclastogenesis,^[67]^ wound healing,^[68]^ and tissue regeneration.^[69]^ This phenomenon plays a pivotal role in cancer.^[70, 71, 72]^ In 1911, it was first hypothesized that cell fusion between tumor cells and cells of the hematopoietic lineage contributes to tumor metastasis. This fusion creates hybrid cells that can grow and spread to new tumors. Recent *in vivo* and *in vitro* studies support this theory.^[13,21]^ These fusions create heterogeneous populations of DPCs, which exhibit enhanced invasiveness,^[8]^ increased metastatic potential,^[13]^ and drug resistance and frequently exhibit cancer stem cell properties.^[73]^ Despite extensive research, the mechanisms underlying this process remain unclear. Notably, although cell fusion in the TME is frequently reported, its actual occurrence probability is low, and only a fraction of DPCs survive, posing challenges for future studies. Compared with studies using transgenic animal models and transgenic tumor cell strains, detecting tumor hybrids in the TME is more challenging and heavily relies on suitable fusion markers. Moreover, distinguishing tumor hybrids from binucleated cells, which may arise because of cytoplasmic division errors, internal replication, or endocytosis, adds complexity.^[74,75]^ Further research is necessary to elucidate the specific regulatory and influencing factors that govern this process.

Cell fusion is a complex and tightly regulated process involving five common steps: Initiation, chemotaxis, membrane adhesion, membrane fusion, and post-fusion reset.^[76]^ Proteins or fusogens that play key roles in this process were identified by bringing the lipid bilayers of the two cell types into direct contact and overcoming the energy barrier to facilitate cell fusion.^[77]^ Actin reorganization, which is orchestrated by interactions between intracellular and extracellular factors, is also crucial in this process.^[78]^ Several key factors involved in the mediation of membrane fusion during normal physiological processes have been implicated in cancer cells, suggesting similar roles in cell fusion dynamics.

Syncytins, phosphatidylserines (PSs), annexins, and glucose-regulated protein 78 (GRP78), known for their role in cytotrophoblast fusion to form syncytiotrophoblasts in the placenta,^[79,80]^ are critical intrinsic factors in cell fusion and are expressed in various cancers, such as endometrial cancer,^[81]^ breast cancer,^[82]^ non-small cell lung cancer,^[83]^ leukemia,^[84]^ and hepatocellular carcinoma.^[85]^ Simultaneously, the syncytin receptor alanine-serine-cysteine transporter 2 (ASCT2) is expressed in prostate cancer cells and promotes tumor growth and metastasis.^[86,87]^ Syncytin-1 expression is correlated with tumor clinical stage, overall survival, vascular invasion, and metastasis, suggesting its prognostic value.^[88,89]^ The mechanism by which syncytin-1 promotes cancer cell fusion remains unclear; however, similarities with retroviral envelope proteins suggest a conserved fusion mechanism.^[90]^ PS is present on the inner layer of the cell membrane^[91]^ and interacts with different receptors and binding proteins, such as T cell immunoglobulin and mucin domain-containing protein 4 (Tim4), brain-specific angiogenesis inhibitor 1 (Bai1), Bai3, Annexin 1, Annexin 5, and CD36. Under conditions such as inflammation, hypoxia, and oxidative stress, PS can be exposed on the surface of cancer cells but not on normal cells, potentially triggering apoptotic pathways and promoting cell fusion.^[92, 93, 94]^ Shabo *et al*. confirmed that the interaction between CD36 and phosphatidylserine plays a role in mediating monocyte-tumor cell fusion.^[95]^ The engulfment and cell motility (ELMO)/dedicator of cytokinesis 1 (Dock180)/rac family small GTPase 1 (Rac1) signaling pathway plays crucial roles in cell migration, phagocytosis, and cytoskeleton reorganization, thereby affecting cancer development by regulating actin dynamics. Annexins, which bind PS, influence processes, such as cancer cell fusion, that are mediated by proteins, such as syncytin 1 and membrane-bound protein A5.^[96, 97, 98]^ In prostate cancer, an increase in muscle cell-induced concentrations of IL-4 and IL-13 and the upregulation of cytokine-induced expression of syncytin 1 and membrane-bound protein A5 were found to promote cell fusion.^[99,100]^ GRP78/binding immunoglobulin protein (Bip) is an endoplasmic reticulum chaperone with calcium-binding and antiapoptotic capabilities. These properties support tumor proliferation, survival, metastasis, and resistance, suggesting their potential as biomarkers for cancer treatment.^[101]^ The interaction of α2M with GRP78 in cancer cells may activate extracellular signal-regulated kinase 1/2 (ERK1/2) signaling, thereby inducing cancer cell fusion.^[102]^ Factors in the TME, including hypoxia, inflammation, viruses, and therapies (radiotherapy and chemotherapy), can induce cell fusion,^[103,104]^ with inflammatory cytokines (TNF-α) enhancing fusion rates through mechanisms involving pathways (Wnt/β-catenin^[105]^ and vascular cell adhesion molecule 1 [VCAM-1]^[106]^). TNF-α-induced cell fusion was attributed to matrix metalloproteinase 9 (MMP9) expression. Inhibiting MMP9 activity with specific inhibitors or suppressing MMP9 expression with dimethylaminotetracycline impaired TNF-α-induced cell-cell fusion rates.^[107]^ These enzymes cleave adhesion molecules from cells, thereby facilitating pathway activation that enhances cell fusion.^[108]^ Viruses induce DPC formation by driving cell fusion. However, the mechanism by which viruses cause cell fusion remains unclear, with two hypothesized pathways: Viral infection activates intracellular fusion progenitors,^[109]^ and viruses act as bridges that connect two cells to promote fusion.^[110]^ Chemotherapy and radiotherapy are also potential inducers of cancer cell fusion, which can lead to polyploid giant cancer cells.^[111]^ However, the direct roles of these factors in cell fusion remain unclear. Cells that successfully survive and express double-positive traits post-fusion undergo a series of processes collectively known as post-hybrid selection to stabilize the chromosomal imbalance.^[112]^

### Phagocytosis

Macrophages are crucial immune cells in the human body that function primarily through the phagocytosis of cellular material and the regulation of tissue repair.^[25,113, 114, 115]^ A significant proportion of DPCs in the TME exhibit characteristics of both tumor cells and macrophages, suggesting that DPC formation *via* macrophage phagocytosis is plausible.^[21,23]^ Macrophages predominantly originate from monocytes, which differentiate from myeloid progenitor cells in the BM under cytokine regulation (IL-34 and CSF1).^[116]^ Some macrophages are derived from the yolk sac or fetal liver, contributing to the tissue-resident macrophage population.^[117,118]^ TAMs are the most abundant immune population in the TME, comprising approximately 50% of hematopoietic cells, and are heterogeneous, ranging from antitumorigenic to protumorigenic.^[119]^ TAM recruitment is facilitated by tumor cell-produced chemokines (chemokine (C-C motif) migand 2 [CCL2], CCL5, and C-X-C motif chemokine ligand 12 [CXCL12]), which attract monocytes for migration to the tumor site. The chemokine (C-C motif) receptor 2 (CCR2)-CCL2 axis is crucial for regulating monocyte migration to metastatic sites and monocyte differentiation into TAMs, thereby promoting cancer growth. Factors such as vascular endothelial growth factor A (VEGFA) and macrophage CSF (M-CSF) are involved in monocyte recruitment and the transformation of TAMs.^[119]^ TAMs primarily impact cancer progression through tumor cell phagocytosis, which is regulated by a complex interaction of “eat me” and “don’t eat me” signals. “Eat me” signals, such as calreticulin and PS, enhance tumor cell phagocytosis by binding to macrophage receptors and activating pathways that direct rearrangement of the actin backbone, thereby driving tumor cell encapsulation and phagocytosis. Conversely, “don’t eat me” signals, including CD47 and PD-ligand 1 PD-L1, inhibit phagocytosis by interacting with receptors, such as signal regulatory protein α (SIRPα), inhibiting myosin II activity and preventing phagosome formation.^[120,121]^ Chou *et al*. identified a class of DPCs formed from incompletely digested phagocytosed tumor cells owing to reverse signaling triggered by TAMs.^[23]^ This process enhances tumor cell survival through antiapoptotic pathways, including increased B-cell lymphoma 2 (BCL-2) expression.^[122]^ Wu *et al*. demonstrated that macrophages can phagocytose tumor cells, alter their functional state, and augment their immunosuppressive capabilities, particularly in glioma tissues.^[21]^ Chou *et al*.^[23]^ utilized a live imaging system to observe the dynamic process of the phagocytosis of tumor cells by macrophages. They employed confocal microscopy to confirm that the deoxyribonucleic acid (DNA) of the phagocytosed tumor cells was located in the cytoplasm of the macrophages rather than in the nucleus, thereby excluding the possibility of cell fusion. The regulation of uncommon cell surface markers, such as CD146, by specific cytokines, such as IL-1β and TNF-α, among TAMs requires further investigation.^[113]^

Moreover, although macrophages are the primary actors in the phagocytosis of tumor cells, other immune cells, such as neutrophils^[114]^ and B cells,^[123]^ also exhibit macrophage-like characteristics. The mechanisms governing DPC formation in these contexts have yet to be fully elucidated,^[124]^ underscoring the diversity and plasticity of TAMs and their diverse implications in cancer biology.

### Immune reprogramming

Immune reprogramming refers to the process by which the functional state, differentiation trajectory, or phenotype of immune cells is reshaped through genetic, epigenetic, metabolic, or environmental interventions.^[125]^ In addition to mechanisms involving cell fusion and phagocytosis that produce DPCs, antigenic stimulation can induce immune reprogramming, leading to the formation of DPCs. This phenomenon has been extensively reported in double-positive T cells.^[16]^

CD4^+^ and CD8^+^ double-positive T cells originate from mature CD4^+^ T cells. These CD4^+^ T cells can re-express CD8^+^ T cells in specific immune microenvironments, such as the gut and TME, in a process that involves altered expression of the transcription factors thymocyte selection-associated high mobility group box protein (ThPOK) and runt-related transcription factor 3 (Runx3). ThPOK normally suppresses CD8 expression, whereas Runx3 promotes CD8 expression,^[126,127]^ leading to the transformation of CD4^+^ T cells into CD4^+^CD8^+^ double-positive T cells by increasing granzyme B (GZMB) and perforin 1 (PRF1) expression.^[35,128]^ Zhu *et al*. confirmed that consumption of Portulaca oleracea L-derived exosome-like nanoparticles can reprogram CD4⁺ T cells into CD4⁺CD8⁺ T cells by downregulating the expression of zinc finger and BTB domain-containing protein 7B (Zbtb7b).^[129]^ Similarly, CD8^+^ T cells can exhibit plasticity and transform into CD4^+^CD8^+^ double-positive T cells under *in vitro* activation conditions.^[35,130]^ The mechanisms underlying these transformations involve the re-expression of opposing co-receptors from specific CD4^+^ or CD8^+^ single-positive T cells, which acquire the phenotypic and functional characteristics of both cell types through T cell receptor (TCR) signaling. Additionally, a novel CD 14^+^CD8^+^ double-positive T-cell type was identified, formed through a cell contact-dependent mechanism involving hepatic stellate cells and other T cells and facilitated by chemokines, such as CXCL12, and extracellular matrix components.^[7]^ Chen *et al*. demonstrated that macrophage-like B cells are not formed through trogocytosis or cell fusion, but rather arise from cancer-induced transdifferentiation.^[18]^ Lad *et al*. demonstrated that IFN-γ in the GBM microenvironment induces the differentiation of immature neutrophils into dendritic-like neutrophils to some extent.^[19]^

In recent years, studies have shown that trogocytosis can enable cells to acquire molecular components that are not inherently expressed by their own lineages.^[131]^ For instance, CD4^+^ T cells can acquire MHC class II molecules from dendritic cells through trogocytosis.^[132]^ Additionally, cancer cells have been reported to acquire markers such as CD4 and CD45 from CD4^+^ T cells during cell-cell contact.^[133]^ However, given that such marker sharing is transient, we have not included trogocytosis-derived DPCs in our classification.

Overall, cells with intrinsic co-expression of lineage markers, such as CD4^+^CD8^+^ T cells, primarily arise through immune reprogramming. After exiting the thymus, they re-express relevant transcription factors in response to external antigenic stimulation, thereby acquiring a double-positive phenotype. Macrophage-like B cells and CD14^+^CD8^+^ T cells are also generated through immune reprogramming processes in response to specific external stimuli, such as exposure to tumor-conditioned media, chemokines, or lipopolysaccharide (LPS). These DPCs are typically diploid and undergo differentiation processes. In contrast, cells with extrinsic co-expression of markers mainly originate from cell fusion or phagocytosis. These cells are polyploid entities that possess mixed genomic and functional characteristics. Through a series of post-hybridization selection processes,^[134,135]^ they can give rise to stably proliferating DPCs, with chromosomal content gradually decreasing over successive passages. The detailed mechanisms of this process have been elucidated in previous reviews.^[112]^ Taken together, these three distinct hypotheses may represent different mechanisms by which various types of DPCs arise, and further research is still needed to elucidate the origins of DPCs. Understanding the roles and developmental pathways of DPCs and whether they are formed through phagocytosis, cell fusion, or immune reprogramming is crucial for developing targeted immunotherapies and improving cancer treatment outcomes.

## Functional characteristics of DPCs in the TME

DPCs perform specific functions on the basis of both tumor and immune cell properties. Tumor hybrid cells typically demonstrate enhanced migration, invasion, and survival. Additionally, immune-tumor DPCs and immune-immune DPCs predominantly exhibit immunosuppressive phenotypes, whereas a small percentage exhibit an immunoregulatory phenotype. The diverse roles of DPCs substantially contribute to the high diversity observed in the TME.

### Enhanced metastatic and invasive properties of DPCs

Gast *et al*. demonstrated that the tumor hybrid cells displayed greater migratory and invasive activities than nonfused cells did.^[13]^ Akhter *et al*. reported that these DPCs exhibited increased invasiveness and upregulated the expression of mesenchymal (zinc finger E-box binding homeobox 1 [ZEB1], SNAIL family transcriptional repressor [SNAIL], TWIST family transcription factor [TWIST], thyroid stimulating hormone subunit beta [THNSB], collagen type XI [COL11]) and epithelial (E-cadherin, N-cadherin, and vimentin) markers, indicating enhanced migratory properties. Additionally, these cells express higher levels of MMP13, suggesting a more aggressive phenotype with increased invasiveness and metastatic potential.^[8]^ Ye *et al*. conducted comprehensive transcriptomic and proteomic analyses that revealed a notable decrease in focal adhesion protein levels in hybrid cells. This finding suggests an impaired adhesion capacity due to compromised adhesion structures. The upregulation of EMT pathway-related genes (cadherin 2 [CDH2], MMP2, MMP3, vimentin [VIM], and secreted phosphoprotein 1 [SPP1]) indicates increased metastatic potential.^[30]^

### Proliferative and tumorigenic capacities of DPCs

Pathways associated with cell proliferation, such as DNA replication and the cell cycle, were notably upregulated in DPCs. Pathways involved in DNA replication, nucleotide metabolism, and pyrimidine metabolism were also enriched. There was also a notable increase in the expression and phosphorylation levels of proliferation markers (proliferating cell nuclear antigen [PCNA] and marker of proliferation Ki-67 [Ki67]), indicating an increased proliferation capacity. With respect to tumorigenicity, Gast *et al*. demonstrated that DPCs exhibit greater environmental adaptability. Under various conditions, DPCs retain their tumorigenic potential and exhibit stronger proliferative activity than non-fused cancer cells because of their enrichment in the bloodstream.^[13]^ Ye *et al*. injected tumor hybrid and tumor cells into mice and reported a considerably increased tumor formation rate in hybrid cells after two months, indicating that tumor hybrid cells possess increased tumorigenic capabilities.^[30]^

**Figure 3:**
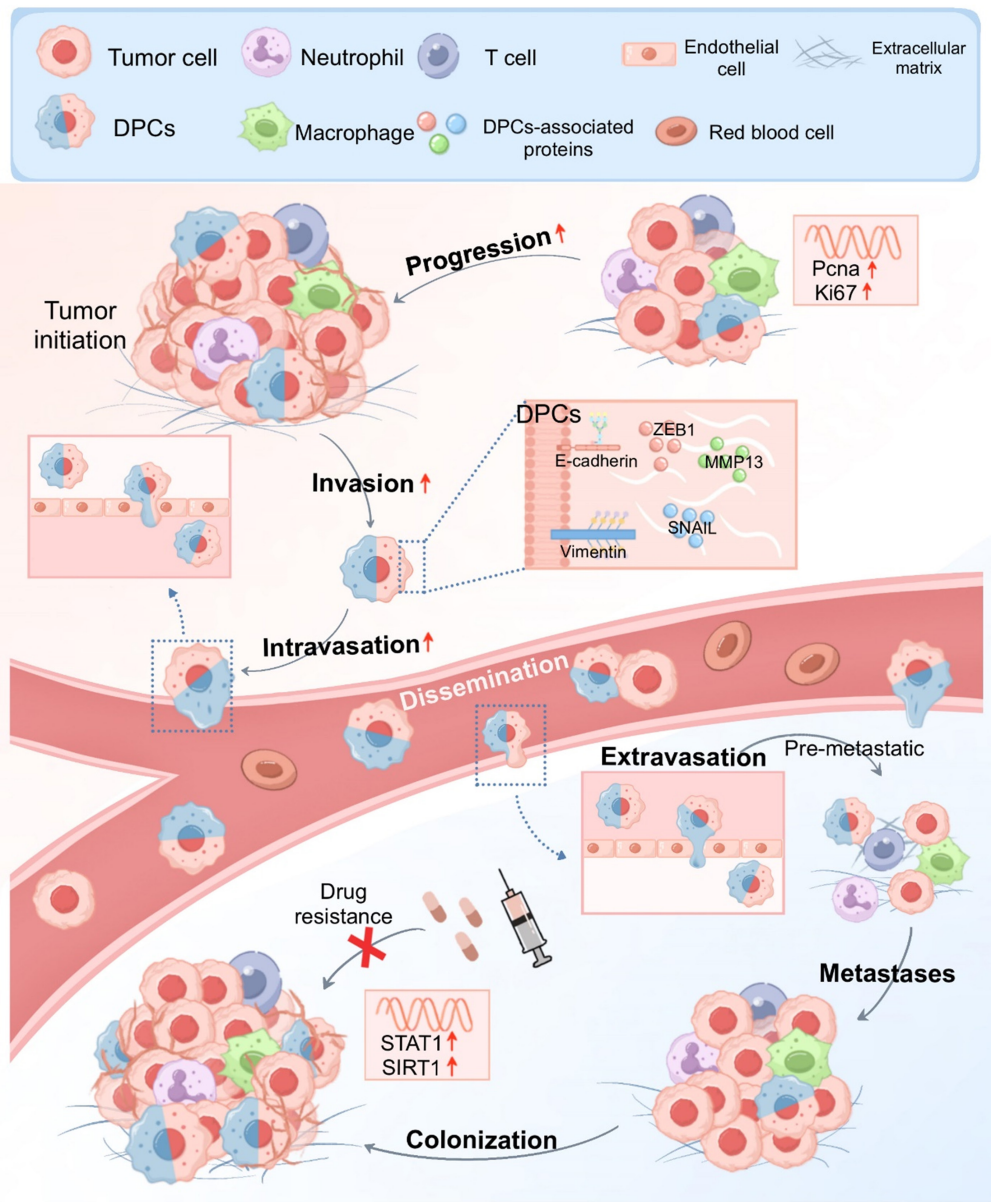
The tumor-associated phenotype of DPCs in the TME. DPCs, especially tumor-associated DPCs (tumor hybrid cells), can metastasize, invade, proliferate, and undergo tumorigenesis. DPCs are characterized by the upregulation of epithelial (E-cadherin and vimentin) and mesenchymal proteins (ZEB1 and SNAIL) on their surface, along with the secretion of MMP13, which facilitates tumor cell invasion and metastasis. Furthermore, DPCs presented upregulated expression of genes associated with proliferation (PCNA and Ki67) and drug resistance (STAT1 and SIRT1). DPCs, double-positive cells; TME, tumor microenvironment; MMP13, matrix metalloproteinase-13; PCNA, proliferating cell nuclear antigen; Ki67, marker of proliferation Ki-67; STAT1, signal transducer and activator of transcription 1; SIRT1, sirtuin 1; ZEB1: zinc finger E-box binding homeobox 1; SNAIL: SNAIL family transcriptional repressor.

Overall, DPCs demonstrated considerably enhanced biological properties, especially metastatic and invasive abilities. These cells exhibit increased migratory and invasive activities *via* the upregulation of mesenchymal markers and matrix metalloproteinases. Additionally, DPCs show enhanced activity in DNA replication and related metabolic pathways, contributing to a higher proliferation rate. DPCs also have increased tumor-forming capacity, reflecting their substantial adaptability and survival in diverse TMEs. However, some studies have reported contrary findings, indicating that further investigation is needed.^[54,58]^ Collectively, these features highlight the complex and unique role of DPCs in tumor biology. DPCs have increased invasiveness and survival, presenting new challenges and opportunities for future research and therapeutic strategies (Figure 3).

### DPCs immunosuppressive phenotype in the TME

Due to the involvement of immune cells, immune-tumor DPCs and immune-immune DPCs exhibit additional specialized immune functions.

Most immune-tumor DPCs exhibit immunosuppression and immune evasion characteristics. Double-positive TAMs (immune-tumor DPCs) are related mainly to M2-type macrophages,^[21,22]^ although a few studies have suggested that they may also display features of M1-type macrophage polarization, albeit not prominently.^[25]^ Extensive analyses have demonstrated a marked upregulation in the expression of M2-type macrophages and tumor-associated markers in these immune-tumor DPCs generated by the phagocytosis of tumors by macrophages, whereas the expression of M1-type markers was notably reduced.^[21,23]^ Specifically, immune-tumor DPCs exhibit elevated levels of chemokines (CXCL12 and CCL22), Fc receptors (Fc fragment of IgE receptor II [FCER2]/CD23 and Fc fragment of IgG receptor IIb [FCGR2B]/CD32), and cytokines (TNF superfamily member 12 [TNFSF12] and interleukin 1 receptor antagonist [IL1RN]). Simultaneously, these cells decreasingly expressed MHC class II molecules (human leukocyte antigen - DR alpha, human leukocyte antigen -DR beta 1 [HLA-DRA, HLA-DRB1]), Fcγ receptor (FCGR1A), and M1-polarization genes (TNF, IL1B, and CD80), which correlated with critical deregulation of cell-cell adhesion, leukocyte migration, antigen processing, and presentation, and cytokine and TCR signaling pathways.^[136,137]^ A cell interaction analysis of double-positive TAMs revealed that increased expression of coinhibitory receptors (PD-1, lymphocyte activation gene-3 [LAG-3], TIM-3, TIM-4, and CD276) by tumor-infiltrating lymphocytes (TILs) exacerbated the dysfunction of CD8^+^ TILs and reduced IFN-γ production. Furthermore, DPCs recruit myeloid cells to the TME by secreting chemokines (CXCL2, CCL2, CCL3, and CCL8) and foster an immunosuppressive environment by inducing the polarization of myeloid cells toward N _2_-type neutrophils or M2-type macrophages *via* CSF1, CSF2, and CSF3. Notably, the transcription factor c-Myc in DPCs binds directly to the promoters of genes associated with M1 and M2 macrophages for immune evasion, thereby increasing their expression and underscoring the pivotal role of cellular Myc (c-Myc) in modulating the immune landscape.^[23]^ DPCs employ a sophisticated immunoregulatory strategy to achieve immune evasion and promote immune checkpoint upregulation (PD-L1, PD-L2, and CD276)^[24]^ while concurrently evading NK cell recognition by maintaining high levels of HLA class I antigen expression.^[8]^

Immune-immune DPCs exhibit diverse and enriched functional characteristics. Immune-immune DPCs play different roles and have opposite functions in immunity against different types of cancer. Zheng *et al*. categorized hepatic tumor-associated double-positive T cells into different subgroups and found that some displayed an immunosuppressive phenotype, whereas others presented immunoregulatory characteristics through single-cell RNA-sequence (seq) and TCR-seq analyses.^[16]^ Specifically, some immunosuppressive subgroups of immune-immune DPCs express high levels of exhaustion markers (cytotoxic T-lymphocyte-associated protein 4 [CTLA4], tumor necrosis factor receptor superfamily member 4/18 [TNFRSF4/18] and lymphocyte activation gene-3 [LAG3]) that enhance tumor immune evasion.^[138,139]^ Schad *et al*. discovered that double-positive T cells derived from CD4^+^ T cells upregulated characteristic cytotoxic genes (GZMB and PRF 1). In contrast, DPCs derived from CD8^+^ T cells exhibit suppressive features *via* the expression of forkhead box P3 (FOXP3).^[35]^ Immune-immune DPCs primarily secrete cytokines that suppress inflammation and display characteristics similar to those of Th2-type T cells. In urological cancers, immune-immune DPCs (CD4^+^CD8^+^ T cells) markedly promote the secretion of Th2-type cytokines (IL-4 and IL-5) rather than Th1-type cytokines.^[43,44]^ These cells also secrete increased levels of the Th2-type cytokines IL-4 and IL-13 to modulate immune responses, possibly facilitating tumor immune evasion in colorectal cancer^[140]^ and melanoma.^[141]^ IL-4 and IL-13 secreted by these DPCs act primarily on helper T and B cells and induce polarization of TAMs toward the M2 type, which is generally associated with anti-inflammatory effects,^[142,143]^ suppression of antitumor immune responses,^[136]^ tumor growth, and spread.^[144]^ Additionally, DPCs secrete IL-5, which plays a role in regulating eosinophils and has substantial protumorigenic effects in breast cancer.^[145,146]^ Compared with monocyte-derived macrophages, DPCs (macrophage-like B cells) more efficiently phagocytose apoptotic cells, suppress T cell proliferation, and induce forkhead box P3^+^ (FOXP3^+^) regulatory T cells to promote breast and ovarian cancer progression and metastasis.^[18]^

### Immunoregulation function of DPCs in the TME

Although most DPCs exhibit an immunosuppressive phenotype, a small percentage of them exhibit an immunoregulatory phenotype. Zheng *et al*. reported that hepatic tumor-associated immune-immune DPCs presented clear activation and cytotoxic characteristics associated with effector T cell activity (fibroblast growth factor binding protein 2 [FGFBP2], GNLY, and granzyme H [GZMH]), cytotoxicity and activation (granzyme K [GZMK], TNFRSF9, and killer cell lectin-like receptor G1 [KLRG1]), and NK T cell (CD70 and killer cell lectin-like receptor D1 [KLRD1) genes, indicating their potential roles in immune surveillance and tumor defense.^[16]^ Notably, most immune-immune DPCs were identified as distinctly clonally expanded in human melanoma and lung cancer using single-cell RNA sequencing and demonstrated tumor reactivity in cytotoxicity assays.^[35]^ The CD14^+^CD8^+^ T cells accumulate within the donor pool in liver allografts, tumor-infiltrating responses, and cirrhotic ascites and exhibit increased turnover, activation, and constitutive immunomodulatory features with high homeostatic IL-10 and IL-2 production and enhanced antiviral/anti-tumor effector function after TCR engagement.^[7]^ iDSCs regulate the migration and positioning of immune cells to the uterine decidua by secreting various angiogenic molecules (vascular endothelial growth factor [VEGF], fibroblast growth factor [FGF], transforming growth factor beta [TGF-β], and platelet-derived growth factor [PDGF]) and participate in immune cell recruitment by expressing chemokines (CCL2, CXCL8, and CX3C chemokine ligand 1 [CX3CL1]) and cell adhesion molecules (intercellular adhesion molecule 1 [ICAM-1] and VCAM-1). They also investigated whether iDSCs could induce cytolysis by activating granzyme-mediated apoptotic signaling pathways. They speculated that these cells could interact with tumor-associated fibroblasts to influence tumor progression.^[61]^

**Figure 4:**
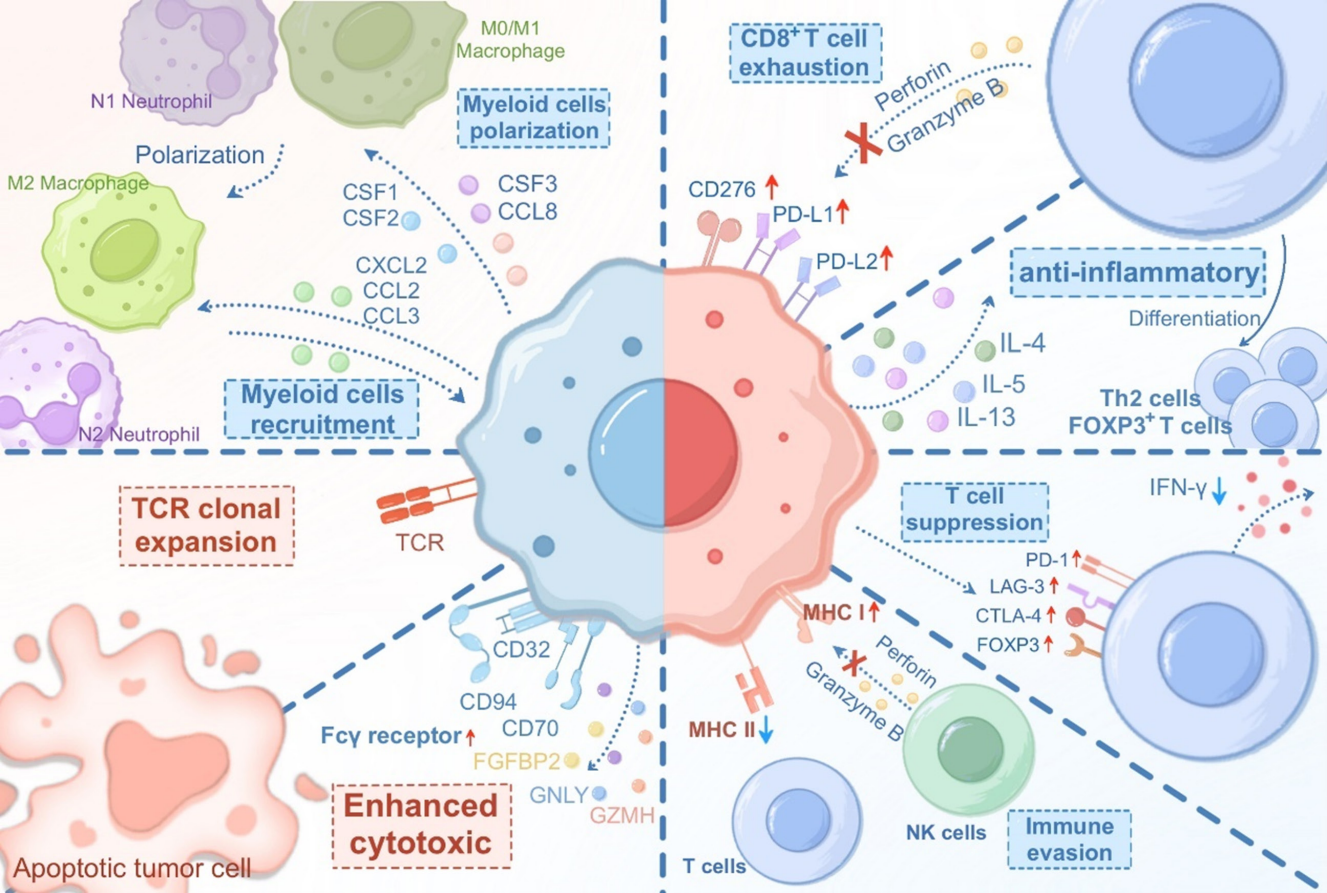
The immunosuppressive and immunoregulatory functions of DPCs. DPCs exhibit functional diversity in modulating the immune response. Immunoregulation (red dashed box): CD4^+^CD8^+^ DPCs demonstrate cytotoxicity through the secretion of FGFBP2, GNLY, and GZMH. Additionally, they exhibit elevated expression of Fcγ receptors on their surface and have the ability to recruit T cells into the TME. DPCs have also been observed to undergo TCR clonal expansion, indicating their active tumor-killing capacity. Immunosuppression (blue dashed box): DPCs can recruit myeloid cells and polarize them into immunosuppressive phenotypes through the secretion of cytokines such as CXCL8, CCL2, CCL3, CCL8, CSF1, CSF2, and CSF3. High expression of PD-L1, PD-L2, and CD276 on DPCs contributes to T-cell exhaustion. DPCs exert anti-inflammatory effects and promote the induction of Th2-type T cells and FOXP3^+^ regulatory T cells by secreting IL-4, IL-5, and IL-13. DPCs can also directly induce the upregulation of co-inhibitory receptors (PD-1, LAG-3, CTLA-4, and FOXP3) on T cells and suppress their production of IFN-γ. DPCs downregulate MHC class II expression while maintaining MHC class I expression on their surface, enabling them to evade immune surveillance. DPCs: double-positive cells; CD: cluster of differentiation; FGFBP2: fibroblast growth factor binding protein 2; GNLY: granulysin; GZMH: granzyme H; Fcγ: Fc gamma; TCR: T cell receptor; TME: tumor microenvironment; CXCL8: C-X-C motif chemokine ligand 8; CCL2: chemokine (C-C motif) ligand 2; CSF1: colony stimulating factor 1; PD-L1: programmed death-ligand 1; PD-1: programmed cell death protein 1; IL: Interleukin; IFN-γ: interferon gamma; MHC: major histocompatibility complex; NK: Natural Killer; FOXP3: forkhead box P3; LAG: lymphocyte activation gene; CTLA: cytotoxic T-lymphocyte associated.

Collectively, the functional characteristics of DPCs in the TME are detailed from four perspectives. Most DPCs with tumor characteristics exhibit enhanced metastatic and invasive properties and increased proliferative and tumorigenic capacities. Additionally, the immunosuppressive phenotype and immunoregulatory role of DPCs have been demonstrated at the molecular level. However, further research is needed on these relevant aspects. These findings are crucial for understanding the intricacies of the tumor immune microenvironment and devising immunotherapeutic approaches for these cells (Figure 4).

## Clinical significance of the DPCs

**Figure 5:**
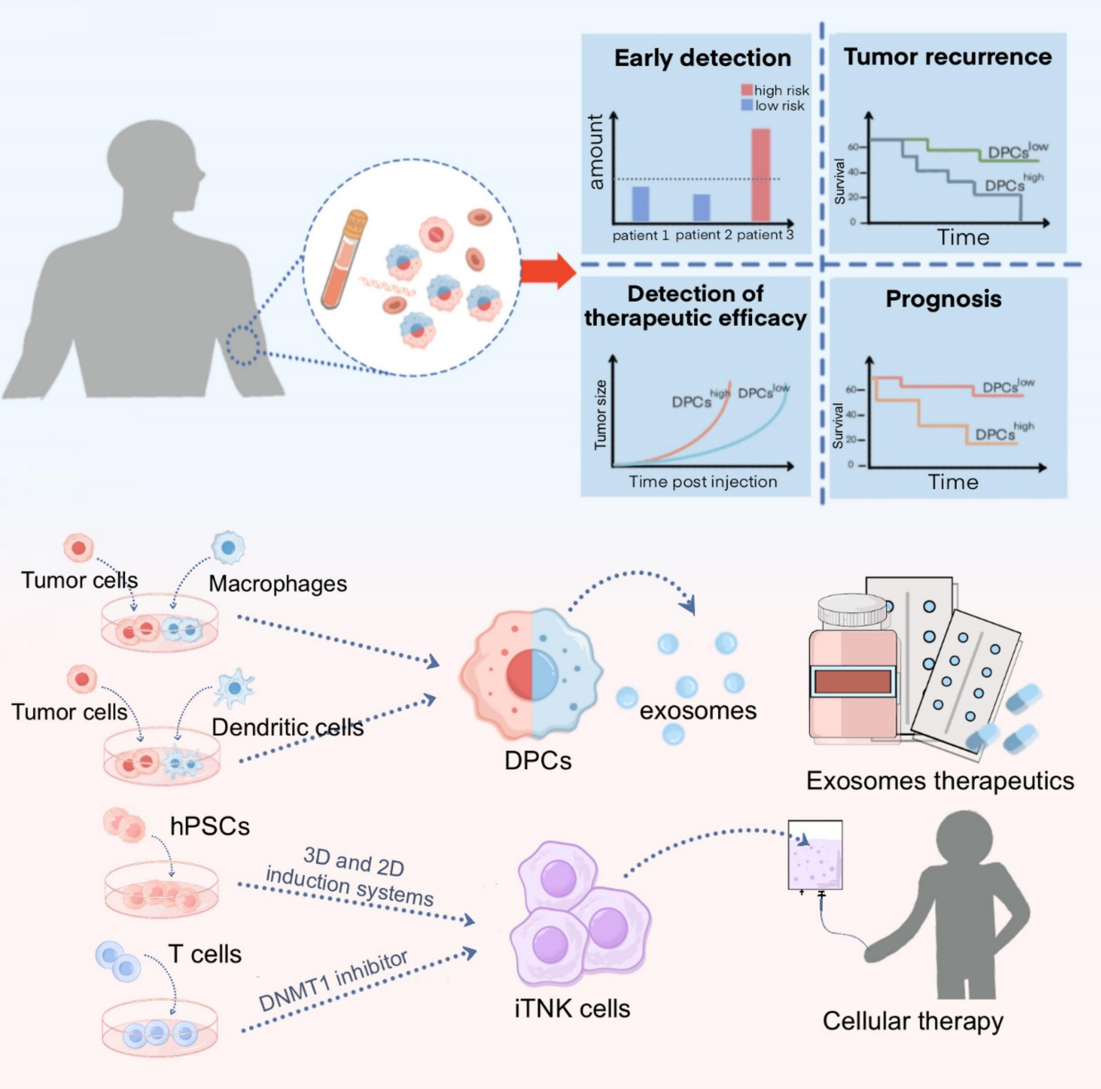
Clinical applications of DPCs. DPCs, especially CHCs, can serve as tumor biomarkers. Measure the levels of DPCs in a patient’s peripheral blood and comparing them to normal values can help patients accurately assess their cancer risk. Additionally, these measurements can provide valuable predictions regarding tumor recurrence, treatment efficacy, and overall prognosis in cancer patients. DPCs can also be generated through the fusion of tumor cells with macrophages or dendritic cells. The exosomes produced by these hybrid cells possess significant therapeutic potential due to their ability to directly activate T cells. A type of DPCs known as iTNK cells can be artificially generated by culturing hPSCs in 2D and 3D induction systems or by applying DNMT1 inhibitors to T cells. Once prepared, these cells are reinfused into patients to exert anti-tumor effects. DPCs: double-positive cells; CHCs: circulating hybrid cells; hPSCs: human pluripotent stem cells; iTNK: induced T-NK; DNMT1: DNA methyltransferase 1.

As a newly identified cellular population within the TME, DPCs hold significant clinical value. The detection of DPCs can provide insights into patient prognosis and treatment efficacy. Moreover, developing novel immunotherapeutic strategies that target DPCs or engineer synthetic antitumor DPCs has become a prominent focus of current research (Figure 5).

DPCs have shown significant relevance to disease stages and potential as biomarkers in tumor immunology, offering new perspectives for cancer diagnosis and treatment.^[29]^ Tumor biomarkers are molecular indicators that reflect the status of a tumor during development and treatment, thereby predicting therapeutic efficacy and prognosis.^[147]^ Biomarkers facilitate the early detection and diagnosis of cancer, monitor disease progression, evaluate therapeutic outcomes, and predict individual responses to specific treatments.^[148]^ The potential of DPCs as biomarkers lies primarily in their ability to mirror the biological behavior and treatment response of tumors. The clinical significance of double-positive CHCs, which are formed by cell fusion, is a focal point in cancer research. Across various cancer types, the number of CHCs is closely associated with tumor aggressiveness and advanced disease stages. A substantial number of these cells can be detected in the peripheral blood of patients with cancer, exceeding the number of traditionally defined circulating tumor cells.^[149]^ The number of CHCs is closely correlated with the clinical stage of tumor progression. In pancreatic,^[13,150]^ ovarian,^[8]^ non-small cell lung,^[151,152]^ oral cavity,^[153]^ breast^[154,155]^ and gastrointestinal cancers,^[156]^ physicians may be able to assess disease progression more effectively, predict a patient’s survival rate, and determine the likelihood of responding to treatment by detecting the number of these cells in a blood sample. Specifically, in ovarian cancer, the proportion of DPCs is significantly greater in patients after chemotherapy than in newly diagnosed patients who have not received treatment, suggesting a possible correlation between disease progression and patient survival rate.^[8]^ Long *et al*. reported that double-positive TAMs are associated with advanced stages of glioma progression and that greater infiltration of double-positive TAMs is correlated with shorter overall survival in patients with GBM.^[25]^

DPCs perform unique functions within the TME, such as promoting cancer cell evasion from immune surveillance and resistance to therapy, making them potential therapeutic targets.^[21,25]^ This foundational understanding will aid in the design of targeted cellular immunotherapy strategies by focusing on immune-stimulating or immune-suppressing molecules expressed by DPCs.^[157]^ Additionally, studies have shown that the use of an anti-V-Set and immunoglobulin domain-containing protein 4 (VISG4) antibody or Thymosin α-1 can reverse the polarization of M2 macrophages, and this therapeutic strategy could be applied to DPCs.^[20,158,159]^ Many researchers have produced DPCs with various functional cellular characteristics for precision cancer therapy.^[160,161]^ The naturally occurring 19305 double-positive T cell receptor (DP-TCR) in CD4^+^CD8^+^ double-positive T cells is a promising therapeutic TCR gene for effective and safe T cell therapy in patients with tumors expressing the New York esophageal squamous cell carcinoma antigen 1 (NY-ESO-1) antigen and HLA-A*02:01.^[162]^ Artificially synthesized DPCs are essential components of cell therapies. Wang *et al*. generated macrophage-tumor hybrid cells. These cells can produce chimeric exosomes, which enter lymph nodes and prime T cell activation in both classical antigen-presenting cell-induced immunostimulatory and unique “direct exosome interactions”.^[160]^ Bao *et al*. produced chimeric exosomes generated from dendritic cell-tumor hybrid cells, allowing direct and robust T-cell activation.^[161]^ The chimeric exosomes secreted by DPCs, as an excellent delivery system and immune activator, allow for the internal packaging of small molecule agonists such as stimulator of interferon genes (STING) agonists.^[161]^ This enables the activation of T cells through antigen presentation while further exerting their antitumor functional effects. Similarly, artificially synthesized exosomes with surface molecules from multiple cell types can also serve as a novel form of cancer immunotherapy. The hybrid exosomes formed by fusing genetically engineered exosomes carrying CD47 from tumor cells with exosomes derived from M1 macrophages exhibit strong tumor-targeting ability and extended circulation time in the bloodstream.^[163]^ Li *et al*. were the first to reprogram T cells into NK cells with a dominance of T cell and NK cell characteristics by deleting Bcl11b.^[164]^ Zhang *et al*. developed a chemically defined protocol enabling sequential induction of mesoderm and hematopoietic lineages in both 2D and 3D systems, generating dual-attribute induced T-NK (iTNK) cells from human pluripotent stem cells (hPSCs) expressing markers of both cytotoxic T and NK cells, which holds promise for advancing immunotherapy.^[165]^ Li *et al*. demonstrated that inhibiting DNA methyltransferase 1 (DNMT1) can reprogram T cells into iTNK cells with potent antitumor activity.^[166]^ However, specific therapeutic approaches targeting DPCs have rarely been studied or validated. With advances in genomic sequencing technologies, such as whole-exome, whole-genome, and single-cell RNA sequencing, researchers can gain a deeper understanding of the mechanisms underlying DPC proliferation, metastatic capabilities, and enhanced drug resistance. These insights will help identify key genes and therapeutic targets.^[167,168]^ For other diseases, artificially synthesized DPCs that combine the characteristics of two different cell types also have exploratory value. Wu *et al*. fused MSCs and platelets to treat cerebral hemorrhage.^[169]^

Overall, DPCs, especially CHCs, can be detected in the peripheral blood of patients with tumors. Therefore, they can be used as biomarkers to predict tumor stage in patients and are correlated with factors such as patient prognosis. Although related studies are limited, immunotherapies designed for the molecular features and immune functions of DPCs have been actively explored.

## Conclusions and perspectives

DPCs are a novel cell subpopulation that expresses characteristic genes of two different cell types. With the advancement of single-cell RNA sequencing technology, an increasing number of DPCs have been reported in the TME. DPCs are highly diverse in the TME and can be categorized into distinct types on the basis of their cellular origins. These cells predominantly arise through processes such as cell fusion, phagocytosis, and immune reprogramming. Tumor-associated DPCs tend to exhibit stronger tumor cell characteristics, such as enhanced invasive and metastatic capabilities as well as drug resistance. DPCs that acquire immune cell phenotypes predominantly display immunosuppressive properties, with a minority showing immunoregulatory functions. DPCs are also present in normal human tissues, where they may provide valuable insights into tumor initiation, progression, and therapeutic response. These cells hold considerable clinical significance, as their levels in patients’ peripheral blood can serve as indicators for tumor staging and prognosis. A wide range of novel immunotherapeutic strategies targeting DPCs are currently under development, and synthetically engineered antitumor DPCs show great promise for future clinical applications.

Previous studies on DPCs have relied primarily on *in vitro* co-culture systems in which immune cells were cultured with fluorescently labeled tumor cells. DPC formation was identified *via* fluorescence imaging, followed by functional assays to investigate DPC characteristics.^[13,23]^ However, this approach significantly disrupted the native behaviors and properties of DPCs within the *in vivo* TME, overlooking their contributions to its complexity. With the rapid advancement of single-cell sequencing technologies, it has become possible to directly profile cell types within the TME and characterize the gene expression landscape of individual cells. This has enabled more direct identification of DPCs.^[25,49]^ Additionally, the development of numerous bioinformatics algorithms now allows for detailed analyses of DPCs, including their differentiation trajectories, functional properties, interactions with other cell types, and spatial distribution through integrative spatial transcriptomics.^[170,171]^ Nonetheless, the preparation of single-cell suspensions for sequencing may introduce technical artifacts, potentially leading to the false identification of some DPCs as contaminants rather than authentic cellular entities.^[172]^ During the data analysis process, researchers need to perform strict quality control, including RNA contamination removal, matrix correction, and double-cell removal. At the same time, it is important to ensure that the gene count and unique molecular identifier (UMI) count of this group of DPCs are within normal ranges and to rule out the possibility that this cell population is enriched only in a few individual samples. It is recommended that future studies employ multi-modal validation following rigorous quality control of single-cell sequencing data. For instance, this could involve integrating high-resolution spatial transcriptomics,^[173,174]^ spatial proteomics,^[175]^ and utilizing immunofluorescence to visualize the co-expression of different marker genes within the same cell on tissue sections.^[176,177]^ Flow cytometry can address the problem of bias caused by cell dissociation in *in situ* detection and analyze the properties of DPCs.^[170]^ Additionally, to minimize technical artifacts and better recapitulate the *in vivo* patient microenvironment, cell sorting by flow cytometry of relevant DPCs followed by functional assays is essential.^[171]^ For novel or low-abundance cells that are difficult to isolate, low-throughput techniques such as Smart-seq can be used to validate high-throughput data analysis results, thereby addressing the challenge of insufficient cell numbers.^[178]^ Nowadays, a range of computational tools has been developed to mitigate contamination, thereby enhancing the precision and credibility of future research.^[179,180]^ In future research, combining spatiotemporal omics and other approaches can further dissect the important roles of DPCs within the TME, clarifying their molecular and functional characteristics.^[181]^

DPCs are a type of cell that has only recently been recognized for playing an important role in the TME. Currently, most research on DPCs remains focused on their identification and characterization. Additionally, DPCs exhibit significant heterogeneity, which has led to a lack of clarity regarding many of the underlying mechanisms, making clinical translation particularly challenging. The current clinical application of DPCs is mainly limited to detecting the content of CHCs in peripheral blood, and as more types of DPCs are being discovered in the TME, future research urgently needs to further unveil the molecular characteristics of these unique DPCs and develop targeted therapies for DPCs. At the same time, leveraging the dual cell characteristics of DPCs to generate more cells with strong anti-tumor effects will be crucial for advancing therapeutic strategies. Overall, as a novel cell type, the characteristics of DPCs require extensive investigation. Future studies should focus on the use of single-cell sequencing technologies in combination with multi-omics approaches to clarify the defining features of DPCs, uncover their heterogeneity, and develop corresponding immunotherapies or targeted treatment strategies after the possibility of technical artifacts is carefully eliminated.
